# Reverse Electrodialysis with Continuous Random Variation in Nanochannel Shape: Salinity Gradient-Driven Power Generation

**DOI:** 10.3390/nano14151302

**Published:** 2024-08-02

**Authors:** Runchen Zhao, Jinhui Zhou, Tianqi Bu, Hao Li, Yanmei Jiao

**Affiliations:** 1 School of Civil Engineering, Nanjing Tech University, Nanjing 211816, China; 2 School of Physical and Mathematical Sciences, Nanjing Tech University, Nanjing 211816, China; 3 School of Mechanical and Power Engineering, Nanjing Tech University, Nanjing 211816, China

**Keywords:** nanochannel shape, nanochannel length, shape amplitude, reverse electrodialysis systems, dimensionless methods

## Abstract

The shape of nanochannels plays a crucial role in the ion selectivity and overall performance of reverse electrodialysis (RED) systems. However, current research on two-dimensional nanochannel shapes is largely limited to a few fixed asymmetric forms. This study explores the impact of randomly shaped nanochannels using dimensionless methods, controlling their randomness by varying their length and shape amplitude. The research systematically compares how alterations in the nanochannel length and shape amplitude influence various system performance parameters. Our findings indicate that increasing the nanochannel length can significantly enhance the system performance. While drastic changes in the nanochannel shape amplitude positively affect the system performance, the most significant improvements arise from the interplay between the nanochannel length and shape amplitude. This compounding effect creates a local optimum, resulting in peak system performance. Within the range of dimensionless lengths from 0 to 30, the system reaches its optimal performance at a dimensionless length of approximately 25. Additionally, we explored two other influencing factors: the nanochannel surface charge density and the concentration gradient of the solution across the nanochannel. Optimal performance is observed when the nanochannel has a high surface charge density and a low concentration gradient, particularly with random shapes. This study advances the theoretical understanding of RED systems in two-dimensional nanochannels, guiding research towards practical operational conditions.

## 1. Introduction

The acquisition of renewable energy to meet the escalating energy demands of human society is a necessary trend in environmental sustainability [1,2]. When effectively harnessed, renewable energy can provide a stable global energy supply, reduce greenhouse gas emissions, and help combat global warming—a critical challenge of the 21st century. Consequently, the use of renewable energy represents a leading trend in global development [3]. Among the diverse array of renewable energy sources, marine energy emerges as a domain with substantial potential yet relatively underdeveloped technology [4]. Salinity gradient energy (SGE) has tremendous potential in being widely applied as a sustainable and eco-friendly energy source due to its unique energy conversion mechanism of directly or indirectly converting Gibbs free energy released from mixing solutions with different concentrations into electricity. One typical SGE comes from the mixing of river water with seawater, which is widely distributed at estuaries all over the world. Three different energy conversion methods, including pressure retarded osmosis (PRO), vapor pressure difference (VPD) energy, and reverse electrodialysis (RED), are most commonly investigated and applied in harvesting SGE [5,6]. Compared to PRO and VPD, RED has a great advantage in directly converting SGE into electricity only through using ion-selective membranes, which avoids mechanical energy losses in PRO or heat dissipation in VPD and thus is also generally named as osmotic energy conversion [7,8].

Numerous studies have demonstrated that the performance of nanofluidic reverse electrodialysis (NRED) systems exhibits significant enhancement compared to traditional RED systems, attributed to higher ion flux and lower fluid resistance [9,10,11,12]. Instead of using traditional ion-selective membranes, this cutting-edge NRED system employs nanofluidic-based membranes with enhanced capability in mass transfer, thereby further boosting the energy conversion efficiency of SGE. Nowadays, the nanofluidic channel stands out as a pivotal component of the NRED system, with the performance enhancement of key parameters such as the open-circuit voltage (OCV), short-circuit current, and power density being constrained by the nanochannel size [13,14]. For the moment, research on energy extraction from salinity gradients through nanofluidic channels predominantly commences from the configuration of these nanochannels [15,16,17,18,19,20,21,22,23,24,25]. Generally, to achieve heightened electrical energy output, traditional methods comprise diminishing the longitudinal dimensions of nanochannels to augment ion flux by reducing ion transport resistance [26]. For instance, Tseng et al. [27] discovered that a nanochannel with a smaller radius or longer channels can improve the ion selectivity and pronounce enhancements in the system performance. Furthermore, altering the nanochannel shapes effectively modifies the size of the electric double layer (EDL) in it. It then subsequently influences the degree of overlap of this EDL. Variations in the ion transport resistance within nanochannels and the overlap degree of the EDL significantly impact the membrane ion selectivity and permeability, which in turn profoundly influence the energy conversion efficiency of RED systems [16,28,29]. Consequently, investigations into nanochannel channel configurations play a crucial role in guiding performance optimization strategies for RED systems.

Nowadays, scholars try to investigate various nanochannel shapes, particularly cylindrical nanochannels, during the studies inspired by biofilm nanochannels. The investigations prefer simulating the functions of biological ion nanochannels [24,30]. This approach involves using cylindrical ways to replicate the regulation of ion currents and gating observed in biological ion nanochannels [30]. On this basis, the scope of research has expanded to conical nanochannels, known for their asymmetric ion transport characteristics [31,32]. When Hsu et al. [33] analyzed conical, bullet-shaped, and trumpet-shaped nanochannels, they discovered smaller nanochannels, especially trumpet-shaped ones, showing a greater EDL overlap and preferable ion selectivity. Changes in the nanochannel length and curvature radius lead to a localized peak in the maximum power output in bullet-shaped nanochannels. In the foundation of two-dimensional straight nanochannel research, scholars have expanded their focus to include two-dimensional layered films in nanofluidic systems [34,35,36,37,38]. In the context of energy extraction from various salt solutions, 2D materials such as two-demensional boron nitrid (BN) and molybdenum dioxide are primarily employed [35]. The strong conversion capability of 2D nanochannels’ surface charge polarity results in energy collection devices with notable cation selectivity [39,40]. Additionally, research on 3D intelligent porous membranes and asymmetric micro/nanochannel structures has also been conducted [41]. However, 2D and 3D nanochannels encounter challenges like fragility, heat resistance, and complexities in large-scale production [42].

The previous literature has extensively examined the characteristics of NRED systems with fixed shapes such as cone-shaped, cylindrical, and bullet-shaped [31,43]. Meanwhile, in practical application scenarios, the channel shape is typically irregular, featuring continuous variations in nanopore morphology. Zhang et al. [44] introduced rectangular units into the nanochannel to induce alterations in the surface roughness by adjusting the width or spacing of artificial nanochannels, hence optimizing the permeation power and energy conversion efficiency. While this method introduces changes in the channel shape, it lacks continuity and randomness to make comparisons with reality. Recognizing the limitations of the existing research, this study employs dimensionless research methods based on the Poisson–Nernst–Planck (PNP) equation and Navier–Stokes (NS) equation to numerically simulate the electrodynamic behavior of nanochannels comprising nanochannels with constantly fluctuating shapes. The investigation systematically explores the impacts of the reservoir salt concentration ratio, asymmetric nanopore fluctuation amplitude, asymmetric nanopore length, and surface charge density on the transfer number of the cation, OCV, resistance, maximum power output, and maximum energy conversion efficiency. Moreover, the comprehensive impact factors of these variables on the parameters of the energy conversion performance of the NRED system are also thoroughly investigated. The findings of this research can serve as a theoretical foundation for advancing the development of NRED systems under practical operating conditions.

## 2. Mathematical Model

### 2.1. Numerical Model

**Figure 1:**
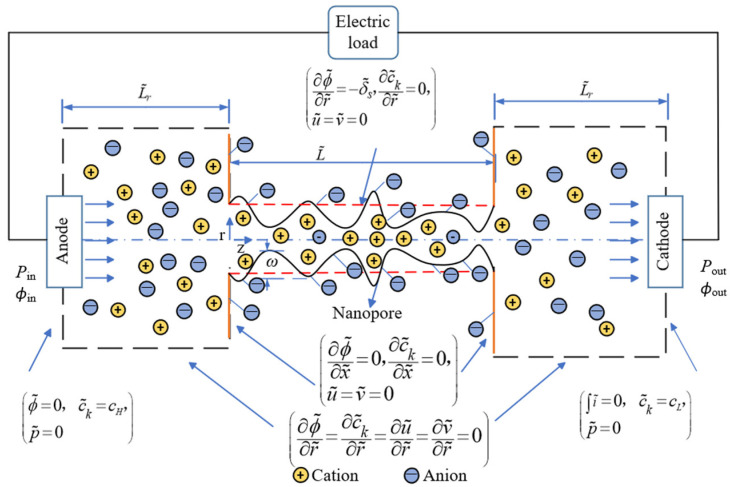
Schematic diagram of NRED system of a random nanochannel shape. The surface of the nanochannel carries negative charges, with arrows representing the direction of ion flow, *ω* representing the shape amplitude of the nanochannel, $\tilde{L}$ representing the dimensionless length of the nanochannel, and systematic boundary conditions marked on both sides of the storage tank and the nanochannel.

As depicted in a typical energy conversion system of NRED in Figure 1**.**, an axisymmetric nanochannel, featured with varied radii along the axial direction, bridges two reservoirs at each end of the nanochannel. The analysis utilized the Navier–Stokes (NS) equation and Poisson–Nernst–Planck (PNP) equation. It is significant to note that the study excludes chemical reactions and electrode polarization phenomena [43]. 

To facilitate numerical calculations and comparisons, a set of characteristic parameters are defined for scaling the governing equations: length *L_ref_* = *a*; concentration *c_ref_* = *c*_0_ with *c*_0_ representing the low solution concentration; relative permittivity *ε_r,ref_* = *ε_r,_*_0_ with *ε_r,_*_0_ representing the dielectric constant at *T* = *T*_0_; diffusion coefficient *D*_1,*ref*_ = *D*_1,0_, *D*_2,*ref*_ = *D*_2,0_ with *D*_1,0_ and *D*_2,0,_ respectively, representing the diffusion coefficients of cations and anions at *T* = *T*_0_; potential *ϕ_ref_* = *RT_ref_*_/_*F* with *R* representing the universal gas constant and *F* representing the Faraday constant; electric strength *E_ref_* = *ϕ_ref/_L_ref_*; fluid viscosity *μ_ref_* _=_ *μ*_0_; charge density *σ_ref_* = *ε*_0_*ε_r,ref_ϕ_ref_*/*L_ref_* with *ε*_0_ representing the vacuum dielectric constant; velocity *u_ref_* = (*ε*_0_ *ε_r,ref_ ϕ_ref_*/*L_ref_*)*E_ref_*; pressure *p_ref_* = *μ_ref_* (*u_ref_*/*L_ref_*); electric current density *i_ref_* = *F u_ref_ c_ref_*; and electric conductivity *τ_ref_* = *i_ref_ L_ref_*/*ϕ_ref_*. Additionally, dimensionless numbers are defined as Reynolds number Re = *ρu_ref_ L_ref_*/*μ_ref_* with *ρ* representing the fluid density, Peclet number of cation Pe_1_ = *u_ref_ L_ref_*/*D*_1_ with *D*_1_ representing the diffusion coefficient of the cation K^+^, Peclet number of anion Pe_2_ = *u_ref_ L_ref_*/*D*_2_ with *D*_2_ representing the diffusion coefficient of the anion Cl^−^, and Pe*_T_* = *ρu_ref_L_ref_Cp*/*k* with *Cp* and *k,* respectively, representing the specific capacity and thermal conductivity. The dimension groups introduced are detailed in Table 1. Figure 1 illustrates the NRED system powered by a concentration gradient. This system comprises negatively charged nanochannels with a shape amplitude labeled *ω* and a dimensionless length $\tilde{L}$, flanked by large cylindrical reservoirs. Each reservoir is filled with saline water, and anode and cathode electrodes are placed at the terminals to detect the potential and current flow. Nanochannel shapes are randomly generated, influenced by both the nanochannel length and shape amplitude. Ion movement within the nanochannel is driven by the concentration gradient of the salt solution across the nanochannels. 

The control equation, derived from the dimensionless parameters, is as follows:

(1) $${\tilde{\nabla }}^{2}\tilde{\phi }=-\frac{1}{2}(\kappa a{)}^{2}\sum z_{k}{\tilde{c}}_{k}$$

(2) $$\mathrm{Pe}(\tilde{u}\cdot \tilde{\nabla }c_{k})={\tilde{\nabla }}^{2}c_{k}+\tilde{\nabla }\cdot (z_{k}c_{k}\tilde{\nabla }\tilde{\phi })$$

(3) $$\tilde{\nabla }\cdot \tilde{u}=0$$

(4) Re(u˜⋅∇˜u˜)=−∇˜p˜+∇˜2u˜−12(κa)2∇ϕ˜∑zkc˜k

In the equation, ϕ is the electric potential. *κ* is the reciprocal of the Debye length, *κ* = [(2*C*_0_*F*^2^)/(*ε*_0_*ε_r_RT*)]^1/2^. *c_k_* and *z_k_* are the concentration and valence, respectively. 

The subscript *k* is denoted as 1 or 2, representing ionic specie K^+^ or Cl^−^, respectively. *C*_0_, *F*, *R,* and *T* are the concentration of the electrolyte solution, faraday constant, universal gas constant, and temperature, respectively. *ε*_0_, *ε*_r,_ *p*, and *u* are the permittivity of the vacuum, the dielectric constant, pressure, and velocity of the fluid, respectively.

Additionally, the current density, a crucial dimensionless parameter, is defined as follows:

(5) $$\tilde{i}=\tilde{u}\sum z_{k}{\tilde{c}}_{k}-\sum z_{k}\frac{1}{\mathrm{Pe}}\tilde{\nabla }{\tilde{c}}_{k}-\tilde{\nabla }\tilde{\phi }\sum z_{k}^{2}\frac{1}{\mathrm{Pe}}{\tilde{c}}_{k}$$

The cation transfer number, a key performance metric for assessing ion selectivity within a nanochannel, is defined as

(6) $$t_{+}=\frac{I_{+}}{I_{+}+|I_{-}|}$$

Here, $I_{+}$ and $I_{-}$ denote the ion currents generated by the flow of cations and anions, respectively. The cation transfer number within the nanochannel ranges from 0 to 1. Values from 0 to 0.5 suggest anion selectivity, while 0.5 to 1 indicate cation selectivity.

The open-circuit voltage in the system is defined as follows:

(7) $$\Phi_{OC}=IR_{e}=I(R_{channel}+R_{load})$$

where *R_channel_* is the nanochannel resistance, *R_load_* is the load resistance in the circuit, and *R_e_* is the total system resistance.

Power generation reflects the RED system’s capacity and is linked to the open-circuit voltage.

(8) $$P=\Phi_{OC}^{2}\frac{R_{\mathrm{load}}}{R_{e}^{2}}$$

Maximum power is attained at half the open-circuit voltage:

(9) $$P_{\max}=\frac{I\Phi_{OC}}{2}=\frac{\Phi_{OC}^{2}}{4R_{e}}$$

Energy conversion efficiency is a metric for measuring a system’s effective energy conversion and assessing its performance. This efficiency is defined as the ratio of the electrical energy recovered from the system to the mixed Gibbs free energy. The energy conversion efficiency at maximum power generation is calculated as follows:

(10) $$\eta_{\max}=\frac{{\left(2t_{+}-1\right)}^{2}}{2}$$

The corresponding values of the constants are as follows, including *F* = 9.649 × 10^4^ C/mol, *T* = 298 K, *R* = 8.314 J/K mol, *ρ* = 1000 kg/m^3^, *μ* = 1 × 10^−3^ Pa s, *ε_r_* = 80, and *ε_0_* = 8.854 × 10^−12^. It should be noted that the solution used in the study is the KCl solution, so the corresponding parameters are as follows: $D_{Cl^{-}}$ = 1.957 × 10^−9^ m^2^/s, $D_{k^{+}}$ = 2.032 × 10^−9^ m^2^/s, $z_{Cl^{-}}$ = −1, and $z_{K^{+}}$ = 1.

In addition, the dimensionless dimensions of the nanochannel and reservoir are $\tilde{a}$ = 1 and $\tilde{L}$ = 5. The values of the dimensionless group set used for calculation in the table are $\phi_{ref}$= 2.57 × 10^−2^ V, $u_{ref}$= 9.34 × 10^−2^ m/s, *p_ref_* = 1.87 × 10^4^ Pa, and *δ_ref_* = 3.64 × 10^−3^ C/m^2^ when the solution in the low concentration area is 1 mM.

### 2.2. Boundary Conditions

Different boundary conditions are used to achieve the open-circuit voltage ${\tilde{\phi }}_{oc}$, short-circuit current ${\tilde{I}}_{sc}$, as well as the voltage–current $\tilde{\phi }-\tilde{I}$ curve. As depicted in Figure 1, the potential at the boundaries is always set as zero. When the current at the boundary of the anode, namely $\int_{A}\tilde{i}$, is set as zero, the induced potential at this boundary is precisely the OCV ${\tilde{\phi }}_{oc}$. In contrast, when the potential at the boundary of the cathode is set as zero, the induced current is precisely the short-circuit current ${\tilde{I}}_{sc}$. The corresponding $\tilde{\phi }-\tilde{I}$ curve can be consequently obtained by setting the potential at the boundary of the cathode as $\tilde{\phi }={\tilde{\phi }}_{0}(0<{\tilde{\phi }}_{0}<{\tilde{\phi }}_{oc})$. All the other relevant boundary conditions are elaborated in Figure 1. 

## 3. Results and Discussions

This study utilized COMSOL Multiphysics software 6.1 for the numerical simulations, integrating four modules within the model. The electrostatic module calculates the potential, the laminar flow module determines the fluid velocity field, and two separate dilute material modules ascertain the concentration distribution of the anions and cations. The model adopts an axisymmetric form, and the nanochannel shape is generated by the internal random function of COMSOL Multiphysics, characterized by its peaks and valleys. The final shape is determined by the nanochannel length and shape amplitude. Additionally, for calculating the flow potential, this study adopted Jiao et al.’s [43] method, applying a boundary condition of zero output current at the nanochannel outlet. This approach circumvents reservoir size limitations. 

**Figure 2:**
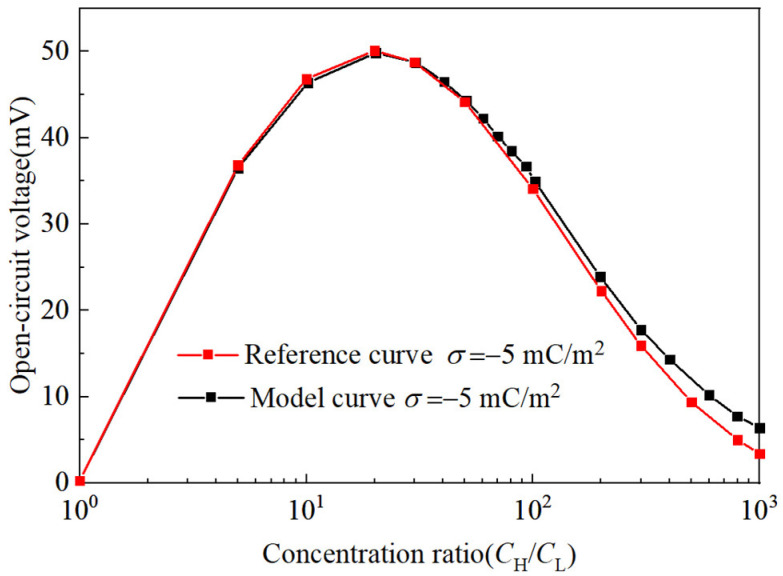
Comparison chart between model curve and reference curve.

Additionally, the model underwent grid independence analysis and validation. Using a discrete model with over 20,000 domain units (refer to Table 2), it was found that exceeding this number of units did not significantly alter the open-circuit voltage results. The model’s OCV performance curve, as a function of the concentration ratio, was compared with the performance reference curve from the relevant literature [45]. The trends of both curves were largely consistent, as depicted in Figure 2. 

### 3.1. The Performance Impact of Nanochannel Length and Shape Amplitude

**Figure 3:**
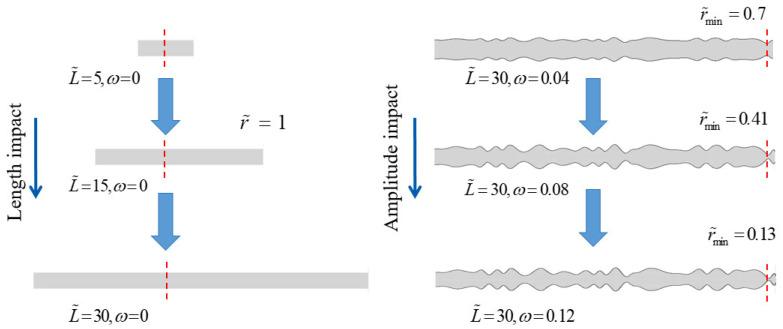
The left transformation represents nanochannel dimensionless length changes of 5, 15, and 30, while the right transformation represents nanochannel shape amplitude changes of 0, 0.04, 0.08, and 0.12.

**Figure 4:**
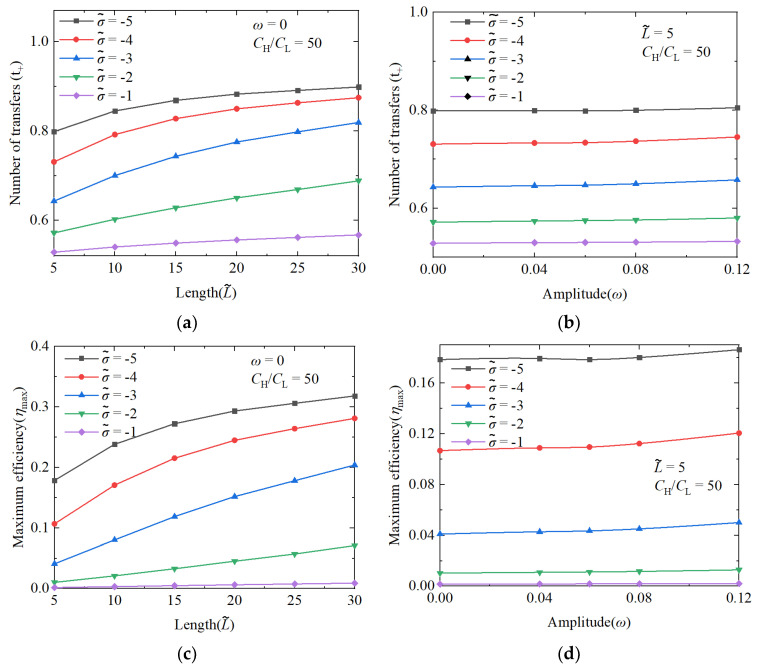
The changes in cation transfer number with nanochannel length and shape amplitude (**a**,**b**), and the changes in maximum energy conversion efficiency with nanochannel length and shape amplitude (**c**,**d**). The nanochannel is a straight nanochannel with a dimensionless surface charge density of −5 and a volume concentration ratio of 50 at both ends of the nanochannel.

As demonstrated in Figure 3, we systematically studied the effects of the nanochannel length and shape amplitude on the performance of an NRED system at varying nanochannel surface charge densities. Figure 4a illustrates that the transfer number of the cation is proportional to the increasing nanochannel length. However, despite the data indicating enhancement, this occurs at a decelerating rate due to the impact of the nanochannel surface charge density. Within the dimensionless surface charge range of −1 to −5, a monotonic increase in the transfer number of the cation was observed with an increasing length at a dimensionless surface charge density of −3. Conversely, at a dimensionless surface charge density of −4, the increase in the transfer number of the cation exhibited a parabolic pattern characterized by slow growth. In contrast, changes in the nanochannel shape amplitude did not significantly enhance the number of cation transfers, as depicted in Figure 4b, primarily highlighting the performance enhancements resulting from the increased nanochannel surface charge density. 

The variation in ion selectivity within the nanochannel, as indicated by the cation transfer number, is closely linked to the extent of charge separation within the EDL of the nanochannel. Prolonging the movement time of ions within the nanochannel through an increase in the nanochannel length facilitates complete separation between the cation and anion ions within the EDL, thereby significantly enhancing the nanochannel ion selectivity. Furthermore, ion selectivity within the nanochannel is also influenced by the degree of overlap between the double electrical layers present within the nanochannel. Fluctuations in the nanochannel radius resulting from changes in the nanochannel shape amplitude can impact the degree of overlap between the EDL within the nanochannel. However, due to the stochastic nature of alterations in the overlap degree, the increase in ion selectivity within the nanochannel is not obvious. 

#### 3.1.1. The Performance Impact of Nanochannel Length

**Figure 5:**
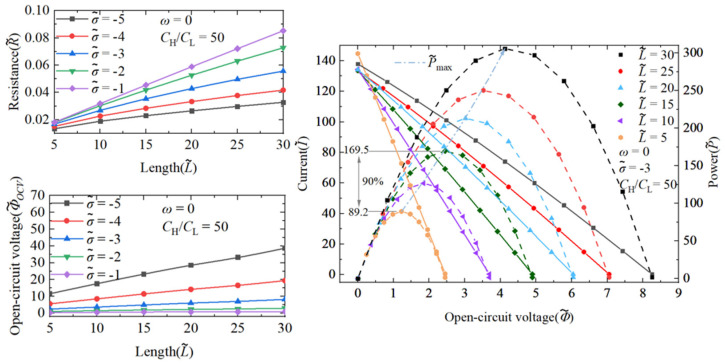
The variation in open-circuit voltage and resistance with nanochannel length. The variation in current and power with voltage is represented by a solid line, while the variation in power with voltage is represented by a dashed line. The nanochannel is a straight nanochannel, with a dimensionless surface charge density of −5 and a volume concentration ratio of 50 at both ends of the nanochannel.

Energy conversion efficiency and power generation are crucial parameters for assessing the performance of NRED systems in terms of permeation power generation. Therefore, it is imperative to investigate how these metrics vary under different nanochannel lengths and shape amplitudes. The increase in the nanochannel length significantly improves the performance of the system. The enhancement of the maximum energy conversion efficiency primarily arises from improvements in the ion selectivity of the nanochannel. As illustrated in Figure 4c,d, the energy conversion efficiency exhibits a consistent correlation with the cation transfer number. In Figure 5, under a concentration gradient of 50 and a dimensionless surface charge density of −3, the dimensionless length increases from 5 to 15, leading to a 90% increase in the maximum generation power performance.

Under conditions where the concentration gradient is set at 50 and the surface charge density of the dimensionless nanochannel ranges from −1 to −5, both the resistance and open-circuit voltage of the system exhibit ohmic behavior as the nanochannel length increases. It is notable that the surface charge density significantly influences the degree of charge separation within the EDL. Consequently, at low surface charge densities, an elongated nanochannel may result in weak electrostatic forces within the nanochannel, leading to the inadequate separation between positive and negative ions. This deficiency weakens the repulsive force of the nanochannel to co-ions and severely hinders counter-ions’ movement. As a result, the nanochannel resistance experiences a more pronounced increase at low surface charge densities.

#### 3.1.2. The Performance Impact of Nanochannel Shape Fluctuation Amplitude

**Figure 6:**
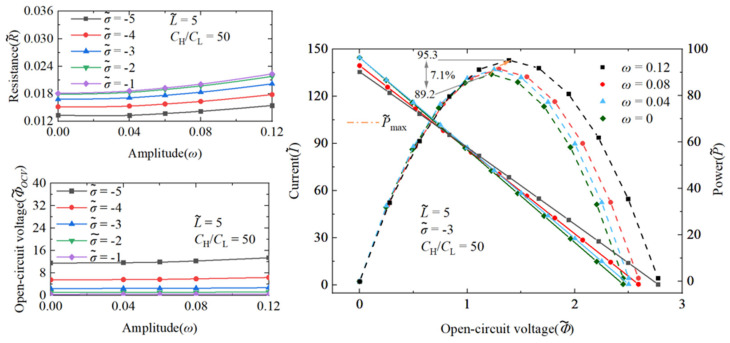
The variation in open-circuit voltage and resistance with the amplitude of random nanochannel shape fluctuations. The variation in current and power with voltage is represented by a solid line, while the variation in power with voltage is represented by a dashed line. The dimensionless length of the nanochannel is 5, the dimensionless surface charge density inside the nanochannel is −5, and the volume concentration ratio on both sides of the nanochannel is 50.

As distinguished from the performance enhancement attributed to the nanochannel length, the influence of the shape amplitude on the energy conversion efficiency and power generation is found to be negligible. Figure 6 illustrates that, under a concentration gradient of 50 and a dimensionless surface charge density of −3, increasing the nanochannel shape amplitude from 0 to 0.12 results in only a marginal 7.1% rise in the maximum power generation. With the difference in the impact of the nanochannel length on the system performance, the shape amplitude does not significantly affect the nanochannel resistance. This lack of effect is complicated and linked to the stochastic nature of the overlap degree of double electric layers within the nanochannel. The reduction in the overlap degree of local double electric layers does not substantially alter the overall ion selectivity. 

#### 3.1.3. Comparison of Performance Effects of Nanochannel Length and Shape Fluctuation Amplitude

**Figure 7:**
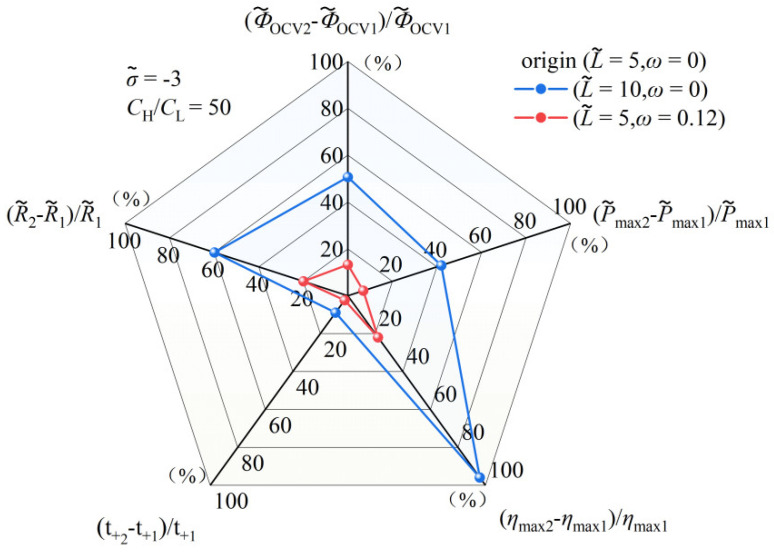
Set the state with a dimensionless length of 5 and a random shape fluctuation amplitude of 0 as the origin, and each axis represents the performance increase in the open-circuit voltage, cation transfer number, maximum power generation, maximum energy conversion efficiency, and nanochannel resistance, respectively. The dimensionless charge density inside the nanochannel is −3, and the volume concentration ratio on both sides of the nanochannel is 50.

To facilitate a more comprehensive comparison of the distinct performance implications arising from variations in the nanochannel length and shape amplitude, a detailed analysis of their respective impacts on the performance of the nanofluidic system is meticulously illustrated in Figure 7. The coordinate origin in this graph corresponds to a dimensionless length of 5 and a shape amplitude of 0. By independently varying the dimensionless length and shape amplitude, the graph compares the maximum performance enhancement achieved by each factor. Figure 7 indicates that while both the dimensionless length and shape amplitude of the nanochannel consistently enhance the performance of the nanofluidic reverse electrodialysis system, the increase due to the length change is significantly more pronounced than that due to the shape amplitude change. Notably, among all the observed performance improvements, the increase in the maximum energy conversion efficiency is the most substantial.

### 3.2. The Superimposed Effect of Nanochannel Length and Shape Amplitude 

**Figure 8:**
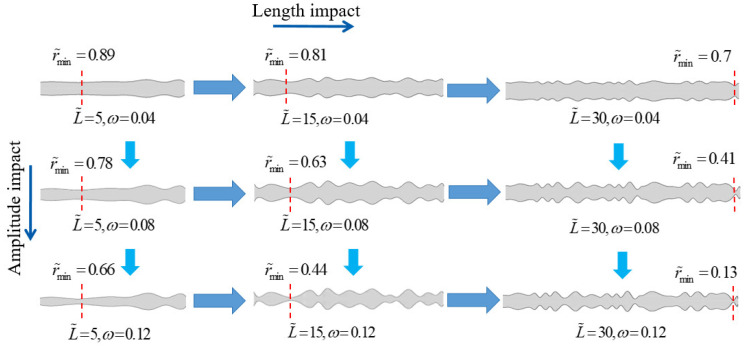
Horizontal transformation represents changes in nanochannel dimensionless lengths of 5, 15, and 30, while vertical transformation represents changes in nanochannel shape amplitudes of 0, 0.04, 0.08, and 0.12.

**Figure 9:**
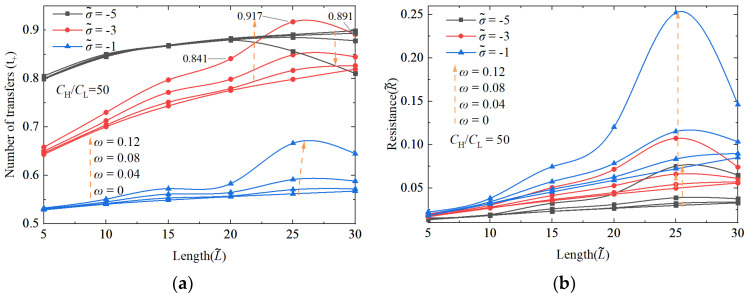

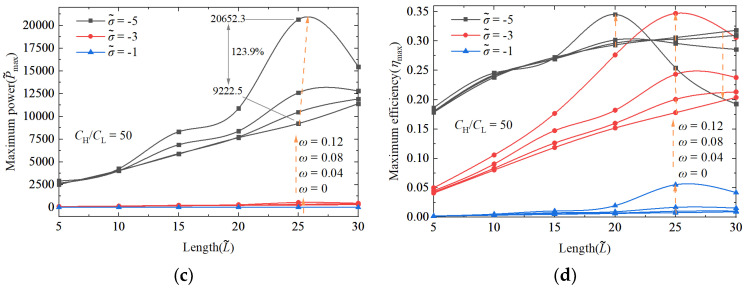
Cationic transfer number (**a**), resistance (**b**), maximum generating power (**c**), and maximum energy conversion efficiency (**d**) change with dimensionless length. The variation amplitude of the nanochannel shape is 0, 0.04, 0.08, and 0.12, respectively, and the volume concentration ratio of solution on both sides of the nanochannel is 50. The dimensionless surface charge densities are −1, −3, and −5, respectively.

In horizontal transformation, Figure 8 illustrates the changes in the nanochannel are 5, 15, and 30. In vertical transformation, the changes in the nanochannel shape amplitudes are 0, 0.04, 0.08, and 0.12. Through the single-factor analysis of the nanochannel length and shape amplitude, it is evident that the nanochannel length exerts a more pronounced influence on the system performance. Further exploration into the combined impact of the nanochannel length and shape amplitude reveals the significance of the nanochannel shape on the permeation power generation performance of NRED systems. Figure 9 illustrates the introduction of the shape amplitude as a factor in the study of the maximum power generation and maximum energy conversion efficiency with respect to the nanochannel length. Local effects manifest in system performance metrics as the length varies, with optimal points emerging under these effects. For instance, in Figure 9c, as the dimensionless nanochannel length ranges from 0 to 30 with a concentration gradient of 50 and dimensionless surface charge density varying from −1 to −5, the maximum power generation peaks near a dimensionless nanochannel length of 25. With a surface charge density of −5, as *ω* changes from 0 to 0.12, there is a notable increase of 123.9% in the maximum power performance.

Similarly, within the same range of nanochannel length variation, the maximum energy efficiency also exhibits optimal performance points. The curve for the low surface charge aligns closely with a dimensionless length of 25, while the high charge density curve peaks at a dimensionless length of 20 (Figure 9d). The cationic transfer number primarily influences the maximum energy conversion efficiency, providing insights into the ion behavior within the nanochannel. In instances where the surface charge density of the nanochannel is low, an increase in the EDL overlap due to the shape amplitude effectively mitigates the performance degradation resulting from insufficient charge separation within the weak static electricity-driven double electric layer.

Furthermore, with the increase in the nanochannel length, the shape amplitude elongates the nanochannel, thereby enhancing its reinforcing effect. However, when a longer nanochannel length induces a more pronounced shape alteration, the overlap of EDLs diminishes, leading to a notable decline in the system performance. For instance, as depicted in Figure 9a, when the concentration gradient is set at 50 and the dimensionless surface charge is −3, increasing the shape amplitude from 0 to 0.12 at a dimensionless nanochannel length of 25 results in the transfer number of the cation of 0.917 outperforming the values obtained at dimensionless lengths of 20 and 30 (0.841 and 0.891, respectively). In scenarios characterized by high surface charge densities, the augmentation of the EDL overlap due to shape variations does not significantly enhance the selectivity of nanochannel ions. Conversely, the system’s performance outcomes are primarily dictated by the surface charge density. Specifically, when the concentration gradient is set at 50, the dimensionless nanochannel length is 25, the dimensionless surface charge is −5, and the shape amplitude increases from 0 to 0.12, the transfer number of the cation only experiences a marginal rise from 0.879 to 0.883. 

**Figure 10:**
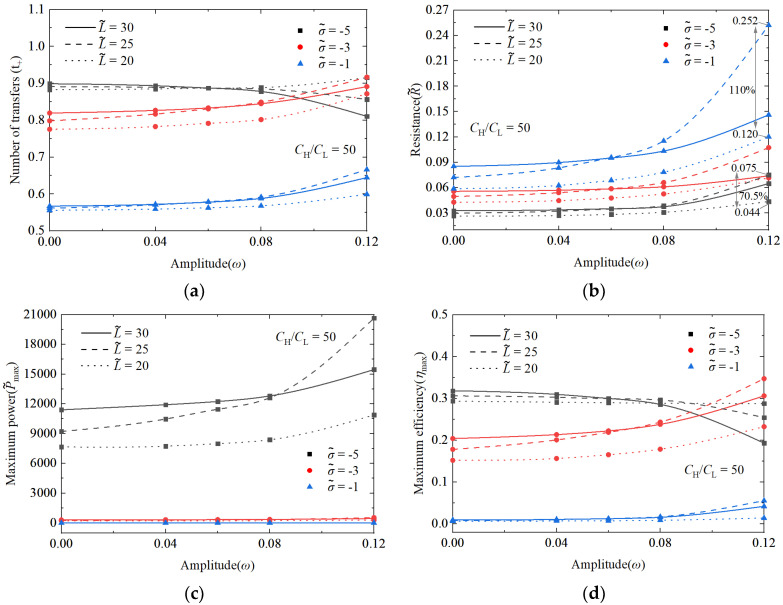
Cationic transfer number (**a**), resistance (**b**), maximum generating power (**c**), and maximum energy conversion efficiency (**d**) change with the amplitude of nanochannel shape. The dimensionless length of the nanochannel changes to 20, 25, and 30, and the volume concentration ratio of the solution at both ends of the nanochannel is 50. The dimensionless surface charge densities are −1, −3, and −5, respectively.

Figure 10 studies the superposition effect in terms of shape amplitude changes, with the same performance changes as Figure 9. It is noteworthy that the superposition effect of the nanochannel length and shape amplitude significantly augments the nanochannel resistance at low surface charge densities, while having a lesser impact on the resistance at high surface charges. As illustrated in Figure 10b, under the conditions of a concentration gradient of 50, dimensionless surface charge density of −1, and shape amplitude of 0.12, an increment in the dimensionless nanochannel length from 20 to 25 leads to a notable 110% rise in the nanochannel resistance. Conversely, for a dimensionless surface charge density of −5, extending the dimensionless nanochannel length from 20 to 25 results in a substantial 70.5% escalation in the nanochannel resistance. Through the above performance analysis, it can be found that the random nanochannel shape of the NRED system, when it belongs to the low concentration gradient, 0.12 shape amplitude, its performance will be better in the dimensionless nanochannel length range of 20 to 25. This set of data is better than the previous study.

### 3.3. The Performance Impact of Randomness Nanochannels on Concentration Ratio

**Figure 11:**
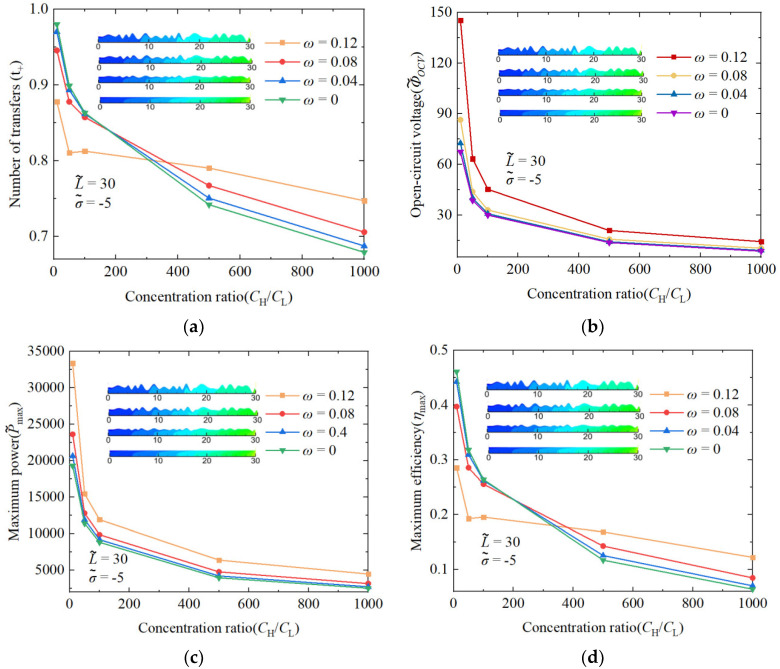
The number of cation transfers (**a**), open-circuit voltage (OCV) (**b**), maximum power generation (**c**), and maximum energy conversion efficiency (**d**) vary with the volume concentration ratio of the solution on both sides of the nanochannel. The dimensionless length of the nanochannel is 30, and the amplitude of the nanochannel shape changes by 0, 0.04, 0.08, and 0.12. The dimensionless surface charge density inside the nanochannel is −5.

In the previous performance study, the concentration gradient of the solution on both sides of the nanochannel remained unchanged at 50, so it is necessary to deeply explore the changes in the various performances of the random nanochannel shape under the change in the concentration gradient, as shown in Figure 11. Figure 11d illustrates a declining trend in the maximum energy conversion efficiency as the solution concentration ratio increases on both sides of the nanochannel. The influence of the nanochannel shape amplitude results in varied performance changes, as indicated by the curve. With more dramatic changes in the nanochannel shape amplitude, the initial maximum energy conversion efficiency is lower, yet the curve’s attenuation is less pronounced. Consequently, a turning point occurs in the concentration ratio range of 200 to 400. Below a ratio of 200, two-dimensional straight nanochannels exhibit a higher energy conversion efficiency. Above 400, nanochannels with greater shape amplitude changes show superior efficiency. This phenomenon is mainly due to the fact that under the high concentration gradient, too strong a solution flow driving force destroys the ion selectivity of the nanochannel, leading to a significant decrease in the transfer number of the cation, while the shape amplitude can prolong the movement time of ions in the nanochannel, so the decrease in the ion selectivity can be slowed down. Figure 11c shows a decrease in the maximum power generation with an increasing concentration ratio of the solution on both sides of the nanochannel. The variation in the maximum power generation is linked to the open-circuit voltage, with its attenuation being closely associated with reduced ion selectivity. 

## 4. Conclusions

This study employs a dimensionless methodology to comprehensively investigate the power generation performance of nanofluidic reverse electrodialysis (NRED) systems with randomly shaped nanochannels. By systematically varying the nanochannel length and random shape amplitude, we have elucidated their distinct impacts on the system performance.

Our findings demonstrate that modifications in the nanochannel length significantly enhance the performance of NRED systems, particularly in nanochannels with a high surface charge density. In contrast, changes in the nanochannel shape amplitude do not lead to substantial performance improvements. At this critical juncture, the primary factor influencing the performance is the surface charge density within the nanochannel.

The interplay between the nanochannel length and shape amplitude results in an optimal performance point for the nanofluidic reverse electrodialysis system. For nanochannels with a low surface charge density, this optimal performance is observed at a dimensionless length of approximately 25. Additionally, variations in the shape amplitude can attenuate the detrimental effects of increased concentration gradients on either side of the nanochannel, thereby enhancing the system performance under low concentration gradient conditions.

This research underscores the importance of optimizing nanochannel dimensions and surface properties to maximize the efficiency of NRED systems. By leveraging the dimensionless approach, we provide a robust framework for understanding and improving the performance of nanofluidic energy conversion technologies.

## Figures and Tables

**Table 1:** Derivation of dimensionless quantization.

Classification	Symbol	Dimensionless Group	Dimensionless Parameter
Length	$L_{ref}$	$L_{ref}=a$	$\tilde{L}=L/a$
Concentration	$C_{ref}$	$C_{ref}=C_{0}$	$\tilde{C}=C/C_{0}$
Potential	$\Phi_{ref}$	$\Phi_{ref}=RT/F$	$\tilde{\Phi }=F\Phi /RT$
Electric strength	$E_{ref}$	$E_{ref}=\Phi_{ref}/L_{ref}$	$\tilde{E}=FaE/RT$
Velocity	$u_{ref}$	$u_{ref}=\varepsilon_{0}\varepsilon_{r}\Phi_{ref}E_{ref}/\mu$	$\tilde{u}=F^{2}\mu au/\varepsilon_{0}\varepsilon_{r}R^{2}T^{2}$
Pressure	$p_{ref}$	$p_{ref}=u_{ref}\mu /L_{ref}$	$\tilde{p}=F^{2}a^{2}p/\varepsilon_{0}\varepsilon_{r}R^{2}T^{2}$
Charge density	$\delta_{ref}$	$\delta_{ref}=\varepsilon_{0}\varepsilon_{r}\Phi_{ref}/L_{ref}$	$\tilde{\delta }=Fa\delta /\varepsilon_{0}\varepsilon_{r}RT$
Electric current density	$i_{ref}$	$i_{ref}=Fu_{ref}C_{ref}$	$\tilde{i}=F\mu ai/\varepsilon_{0}\varepsilon_{r}R^{2}T^{2}C_{0}$
Electric conductivity	$\tau_{ref}$	$\tau_{ref}=i_{ref}L_{ref}/\Phi_{ref}$	$\tilde{\tau }=\mu \sigma /\varepsilon_{0}\varepsilon_{r}RTC_{0}$
Electric resistance	$R_{e}$	$R_{e}=1/\tau_{ref}L_{ref}$	${\tilde{R}}_{e}=\varepsilon_{0}\varepsilon_{r}RTC_{0}aR_{e}/\mu$
Reynolds number	Re	Re=ρurefLref/μ	——
Peclet number	Pe	$\mathrm{Pe}=u_{ref}L_{ref}/D_{k}$	——

**Table 2:** Irrelevance verification. When the dimensionless length is 5, the shape amplitude is 0.12, the concentration ratio of the solutions is 50, and the dimensionless surface charge density is −5, the OCV outputs and corresponding computation times for different grids are as follows.

Given Parameters	$\tilde{L}$	ω	${\tilde{C}}_{H}$	${\tilde{C}}_{L}$	$\tilde{\sigma }$
	5	0.12	50	1	−5
Results					
Number of units	9978	11,084	25,342	37,366	51,534
${\tilde{\Phi }}_{OCV}$	12.7	13.3	13.4	13.4	13.4
Computing time (s)	24	524	1386	1875	2591

## Data Availability

Data will be made available on request.
